# 2022 World Hypertension League, Resolve To Save Lives and International Society of Hypertension dietary sodium (salt) global call to action

**DOI:** 10.1038/s41371-022-00690-0

**Published:** 2022-05-17

**Authors:** Norm R. C. Campbell, Paul K. Whelton, Marcelo Orias, Richard D. Wainford, Francesco P. Cappuccio, Nicole Ide, Bruce Neal, Jennifer Cohn, Laura K. Cobb, Jacqui Webster, Kathy Trieu, Feng J. He, Rachael M. McLean, Adriana Blanco-Metzler, Mark Woodward, Nadia Khan, Yoshihiro Kokubo, Leo Nederveen, JoAnne Arcand, Graham A. MacGregor, Mayowa O. Owolabi, Liu Lisheng, Gianfranco Parati, Daniel T. Lackland, Fadi J. Charchar, Bryan Williams, Maciej Tomaszewski, Cesar A. Romero, Beatriz Champagne, Mary R. L’Abbe, Michael A. Weber, Markus P. Schlaich, Agnes Fogo, Valery L. Feigin, Rufus Akinyemi, Felipe Inserra, Bindu Menon, Marcia Simas, Mario Fritsch Neves, Krassimira Hristova, Carolyn Pullen, Sanjay Pandeya, Junbo Ge, Jorge E. Jalil, Ji-Guang Wang, Jiri Wideimsky, Reinhold Kreutz, Ulrich Wenzel, Michael Stowasser, Manuel Arango, Athanasios Protogerou, Eugenia Gkaliagkousi, Flávio Danni Fuchs, Mansi Patil, Andy Wai-Kwong Chan, János Nemcsik, Ross T. Tsuyuki, Sanjeevi Nathamuni Narasingan, Nizal Sarrafzadegan, María Eugenia Ramos, Natalie Yeo, Hiromi Rakugi, Agustin J. Ramirez, Guillermo Álvarez, Adel Berbari, Cho-il Kim, Sang-Hyun Ihm, Yook-Chin Chia, Tsolmon Unurjargal, Hye Kyung Park, Kolawole Wahab, Helen McGuire, Naranjargal J. Dashdorj, Mohammed Ishaq, Deborah Ignacia D. Ona, Leilani B. Mercado-Asis, Aleksander Prejbisz, Marianne Leenaerts, Carla Simão, Fernando Pinto, Bader Ali Almustafa, Jonas Spaak, Stefan Farsky, Dragan Lovic, Xin-Hua Zhang

**Affiliations:** 1 Special Advisor to the board, and Past president (ex officio), World Hypertension League, Hong Kong, China; 2 Department of Medicine, University of Calgary, Calgary, AB Canada; 3 President-Elect, World Hypertension League, Hong Kong, China; 4 Tulane University School of Public Health and Tropical Medicine, New Orleans, LA USA; 5 Vice President, World Hypertension League, Hong Kong, China; 6 Yale University, New Haven, CT USA; 7 Sanatorio Allende, Córdoba, Argentina; 8 Chair, International Society of Hypertension, Membership Committee, International Society of Hypertension, Colchester, UK; 9 Department of Pharmacology and Experimental Therapeutics and the Whitaker Cardiovascular Institute, Boston University School of Medicine, Boston, MA USA; 10 Past President, British and Irish Hypertension Society, Edinburgh, UK; 11 University of Warwick, WHO Collaborating Centre for Nutrition, Warwick Medical School, Coventry, UK; 12 Resolve to Save Lives, an Initiative of Vital Strategies, New York, NY USA; 13 WHO Collaborating Centre on Population Salt Reduction, Sydney, NSW Australia; 14 The George Institute for Global Health, Department of Epidemiology and Biostatistics, School of Public Health, Imperial College London, St Mary’s Campus, Norfolk Place, Paddington, London, UK; 15 The George Institute for Global Health, University of New South Wales, Sydney, NSW Australia; 16 Wolfson Institute of Population Health, Barts and The London School of Medicine & Dentistry, Queen Mary University of London, London, UK; 17 Department of Preventive & Social Medicine, University of Otago, Dunedin, New Zealand; 18 Costa Rican Institute of Research and Teaching in Nutrition and Health (INCIENSA), Tres Rios, Costa Rica; 19 Officer at Large, International Society of Hypertension, Chair International Society of Hypertension Research and Education Committee, International Society of Hypertension, Colchester, UK; 20 Center for Health Evaluation and Outcomes Sciences, University of British Columbia, Vancouver, BC Canada; 21 International Society of Hypertension Education Lead, Colchester, UK; 22 Department of Preventive Cardiology, National Cerebral and Cardiovascular Center, Osaka, Japan; 23 Advisor Food, Nutrition and Physical Activity in Schools, Pan American Health Organization, Washington DC, MD USA; 24 Faculty of Health Sciences, Ontario Tech University, Oshawa, ON Canada; 25 Chair, Action on Salt, London, UK; 26 Chair, Action on Sugar, London, UK; 27 Chair, World Action on Salt Sugar and Health (WASSH), London, UK; 28 Chair Blood Pressure UK, London, UK; 29 Wolfson Institute of Population Health, Barts and The London School of Medicine & Dentistry, Queen Mary University of London, London, UK; 30 Board member, Director (Sub-Saharan Africa), World Hypertension League, Hong Kong, China; 31 Dean, Faculty of Clinical Sciences, Director Center for Genomic and Precision Medicine, College of Medicine, University of Ibadan, Ibadan, Nigeria; 32 World Stroke Organization Geneva, Geneva, Switzerland; 33 Lead Co-Chair Lancet Commission on Stroke, London, UK; 34 Past President (ex officio), World Hypertension League, Hong Kong, China; 35 Secretary-General, World Hypertension League, Hong Kong, China; 36 Italian Society of Arterial Hypertension, Milan, Italy; 37 Department of Medicine and Surgery, University of Milano-Bicocca and IRCCS, Istituto Auxologico Italiano, Milan, Italy; 38 Past President, World Hypertension League, Hong Kong, China; 39 Division of Translational Neurosciences and Population Studies, Department of Neurology, Medical University of South Carolina, Charleston, SC USA; 40 Treasurer, International Society of Hypertension, Colchester, UK; 41 Health Innovation and Transformation Centre, Federation University Australia, Ballarat, VIC Australia; 42 Department of Cardiovascular Sciences, University of Leicester, Leicester, UK; 43 Department of Anatomy and Physiology, University of Melbourne, Parkville, VIC Australia; 44 Secretary, International Society of Hypertension, Colchester, UK; 45 University College London, NIHR University College London, Hospitals Biomedical Research Centre, London, UK; 46 President, International Society of Hypertension, Colchester, UK; 47 Division of Cardiovascular Sciences, Faculty of Medicine, Biology and Health, University of Manchester, Manchester, UK; 48 Manchester Heart Centre and Manchester Academic Health Science Centre, Manchester University NHS Foundation Trust, Manchester, UK; 49 Chair, International Society of Hypertension Regional Advisory Group – Americas Colchester, Colchester, UK; 50 Department of Internal Medicine, Emory University, Atlanta, Georgia; 51 Director, Coalición Latinoamérica Saludable (CLAS), McKinney, TX USA; 52 Department of Nutritional Sciences, Temerty Faculty of Medicine, University of Toronto, Toronto, ON Canada; 53 Director, WHO Collaborating Centre on Nutrition Policy for Chronic Disease Prevention, Toronto, ON Canada; 54 Advisor, Executive Committee, World Hypertension League, Hong Kong, China; 55 Professor of Medicine, Division of Cardiovascular Disease, State University of New York, Downstate Medical Center, New York, NY USA; 56 President, High Blood Pressure Research Council of Australia, Tuggerah, NSW Australia; 57 Dobney Hypertension Centre, Medical School - Royal Perth Hospital Unit, Royal Perth Hospital Research Foundation, The University of Western Australia, Perth, WA Australia; 58 President, International Society of Nephrology, Brussels, Belgium; 59 Professor of Pathology, Microbiology and Immunology, Professor of Medicine, John L. Shapiro Chair of Pathology, Professor of Pediatrics, Vanderbilt University, Nashville, TN USA; 60 Co-Chair Global Policy Committee, member of the Executive Committee, World Stroke Organization, Geneva, Switzerland; 61 Director of National Institute for Stroke and Applied Neurosciences, Auckland University of Technology, Auckland, New Zealand; 62 Founding Chair, Steering Committee, African Stroke Organization, Ibadan, Nigeria; 63 Institute for Advanced Medical Research and Training and Center for Genomic and Precision Medicine, College of Medicine, University of Ibadan, Ibadan, Nigeria; 64 Past President, Argentine Society of Arterial Hypertension, Buenos Aires, Argentina; 65 Advisor to the Academic Vice-Chancellor, Maimonides University, Buenos Aires, Argentina; 66 Founder and Secretary, Dr Bindu Menon Foundation (India), Nellore, India; 67 Nutritionist, Brazilian Society of Hypertension, Sao Paulo, Brazil; 68 Past President, Brazilian Society of Hypertension, Sao Paulo, Brazil; 69 Full Professor of Internal Medicine and Director of the Faculty of Medical Sciences, State University of Rio de Janeiro, Rio de Janeiro, Brazil; 70 Board member, Director - Eastern European Regional Office, World Hypertension League, Hong Kong, China; 71 Member, Research and Education Committee, International Society of Hypertension, Colchester, UK; 72 Bulgarian League of Hypertension, President of Bulgarian Society of Cardiovascular Imaging, Sofia, Bulgaria; 73 Sofia University, Faculty of Medicine, Center of Cardiovascular diseases, Sofia, Bulgaria; 74 Chief Executive Officer, Canadian Cardiovascular Society, Ottawa, ON Canada; 75 President, Canadian Society of Nephrology, Montreal, QC Canada; 76 President, Cardiology Branch, Chinese Medical Doctors Association, Beijing, China; 77 President, Chilean Society of Hypertension, Santiago, Chile; 78 Advanced Center for Chronic Diseases (ACCDiS), Division de Enfermedades Cardiovasculares, Facultad de Medicina, Pontificia Universidad Católica de Chile, Santiago, Chile; 79 President, Chinese Hypertension League, Beijing, China; 80 The Shanghai Institute of Hypertension, Ruijin Hospital, Shanghai Jiao Tong University School of Medicine, Shanghai, China; 81 President, Czech Society of Hypertension, Prague, Czech Republic; 82 President, European Society of Hypertension, Zug, Switzerland; 83 Charité – Universitätsmedizin Berlin, Institute of Clinical Pharmacology and Toxicology, Berlin, Germany; 84 Chairman of the Board, German Society of Hypertension, Heidelberg, Germany; 85 Past President, Treasurer, High Blood Pressure Research Council of Australia, Tuggerah, NSW Australia; 86 Director, Policy and Advocacy, Heart and Stroke Foundation of Canada, Ottawa, ON Canada; 87 Board member, Hellenic Society of Hypertension, Athens, Greece; 88 National and Kapodistrian University of Athens, Athens, Greece; 89 Treasurer, Hellenic Society of Hypertension, Athens, Greece; 90 Aristotle University of Thessaloniki, Thessaloniki, Greece; 91 Hospital de Clínicas de Porto Alegre, Porto Alegre, Brazil; 92 School of Medicine, Universidade Federal do Rio Grande do Sul, Porto Alegre, Brazil; 93 Chief Program Officer, Hypertension and Nutrition Core Group of India Association for Parenteral and Enteral Nutrition (IAPEN), Maharashtra, India; 94 Director of IAPEN, Maharashtra, India; 95 President, Hong Kong College of Cardiology, Hong Kong, China; 96 Secretary General, Hungarian Society of Hypertension, Budapest, Hungary; 97 Department of Family Medicine, Semmelweis University, Budapest, Hungary; 98 President, Hypertension Canada, Toronto, ON Canada; 99 President, Indian Society of Hypertension, Lucknow, India; 100 Board Member, World Hypertension League, Hong Kong, China; 101 President Elect, Iranian Heart Federation, Tehran, Iran; 102 Professor of Medicine and Cardiology, Director of Isfahan Cardiovascular Research Center, Isfahan, Iran; 103 WHO Collaborating Center, Cardiovascular Research Institute, Isfahan University of Medical Sciences, Isfahan, Iran; 104 Executive Director, InterAmerican Heart Foundation (IAHF), Dallas, TX USA; 105 International Council of Cardiovascular Prevention and Rehabilitation (ICCPR), Markham, ON Canada; 106 Singapore Heart Foundation, Singapore, Singapore; 107 President, Japanese Society of Hypertension, Tokyo, Japan; 108 President, Latin American Society of Hypertension, Buenos Aires, Argentina; 109 Arterial Hypertension and Metabolic Unit, University Hospital, Fundacion Favaloro, Medical Sciences Faculty, University Dr. RG Favaloro, Buenos Aires, Argentina; 110 President, Latin American Society of Nephrology and Hypertension, Innova, Panama; 111 Past President of the Central American and Caribbean Association of Nephrology and Hypertension, Santa Domingo, Dominican Republic; 112 President, Lebanese Hypertension League, Beirut, Lebanon; 113 President, Korean Society of Community Nutrition, Seoul, South Korea; 114 Seoul National University Seoul, Seoul, South Korea; 115 President, Korean Society of Hypertension, Seoul, South Korea; 116 Division of Cardiology, The Catholic University of Korea, Seoul, South Korea; 117 Immediate Past President, Malaysian Society of Hypertension, Selangor, Malaysia; 118 President, Malaysian Society for World Action on Salt, Sugar and Health (MyWASSH), Selangor, Malaysia; 119 Department of Medical Sciences, School of Medical and Live Sciences, Sunway University, Bandar Sunway, Selangor, Malaysia; 120 President, Mongolian Society of Hypertension, Ulaanbaatar, Mongolia; 121 Department of Cardiology, School of Medicine, Mongolian National University of Medical Sciences, Ulaanbaatar, Mongolia; 122 General Director, National Institute of Food and Nutrition Service, Seoul, South Korea; 123 Secretary-General, Nigerian Hypertension Society, Ibadan, Nigeria; 124 Department of Medicine, University of Ilorin, Ilorin, Nigeria; 125 NCD Global Program Leader, PATH, Seattle, WA USA; 126 Chief Executive Officer and Co-Founder Onom Foundation, Onom Foundation, Ulaanbaatar, Mongolia; 127 Secretary General and Founder Trustee, Pakistan Hypertension League, Karachi, Pakistan; 128 Chair of International Society of Hypertension Regional Advisory Group South, and Central Asia, Colchester, UK; 129 Karachi Institute of Heart Diseases, Karachi, Pakistan; 130 President, Philippine Society of Hypertension, Pasig City, Philippines; 131 Department of Medicine, University of the Philippines College of Medicine and St. Luke’s Medical Center, Quezon City, Philippines; 132 Immediate Past President, Philippine Society of Hypertension, Pasig City, Philippines; 133 Faculty of Medicine and Surgery, University of Santo Tomas, Manila, Philippines; 134 President, Polish Society of Hypertension, Gdansk, Poland; 135 Department of Hypertension, National Institute of Cardiology, Warsaw, Poland; 136 Co-Director, Primary Aldosteronism Foundation, Phoenix, AZ USA; 137 Coordinator, Portuguese Society of Paediatric Working Group on Blood Pressure in Children and Adolescents, Lisbon, Portugal; 138 President, Portuguese Society of Hypertension, Lisbon, Portugal; 139 Head, Continuous Professional Development, Saudi Hypertension Management Society, Riyadh, Saudi Arabia; 140 President, Swedish Society for Hypertension, Stroke and Vascular Medicine, Stockholm, Sweden; 141 Associate Professor in Cardiology, Department of Clinical Sciences, Danderyd University Hospital, Karolinska Institutet, Stockholm, Sweden; 142 Chairman, Slovak League against Hypertension, Bratislava, Slovakia; 143 Past President, Serbian Society of Hypertension, Nis, Serbia; 144 Clinic for Internal disease Intermedica Cardiology Department, Hypertensive Centre, Singidunum University, School of Medicine, Belgrade, Serbia; 145 President, World Hypertension League, Hong Kong, China

**Keywords:** Risk factors, Lifestyle modification

## Introduction

This fact sheet and global call to action is aimed at nutrition, hypertension, cardiovascular and other health care clinicians and scientists, and health advocates, as well as the organizations to which they belong. The ‘call’ is to align these audiences with the facts on:

- the burden of disease and key evidence supporting reductions in dietary sodium,
- the consistent recommendations for reducing dietary sodium from unbiased and comprehensive health and scientific reviews,
- the current levels of sodium intake,
- the cost savings expected from reducing high dietary sodium,
- the sources of controversial opinions,
- the current recommended approaches to reduce dietary sodium, and
- how to stay up to date with evidence on how to reduce dietary sodium and the evolving research on the adverse health effects of a high sodium intake.

Health, nutrition, hypertension and cardiovascular organizations, and their members, need to become more engaged and advocate for reductions in dietary sodium, and for a greater priority to be given to high quality research on dietary sodium. The World Hypertension League, Resolve to Save Lives and International Society of Hypertension are committed to support reductions in dietary sodium as a high priority.

## Diets high in sodium (salt, sodium chloride- see Table 1 for equivalents) are associated with a high burden of disease from increased blood pressure, cardiovascular disease (CVD), premature death and disability

- Increased blood pressure (BP) is the leading preventable risk factor for heart disease (heart attack and heart failure), stroke, and kidney failure; and a major contributor to premature death, dementia, disability and health care costs [1–4].

**Table 1:** Sodium is largely ingested as sodium chloride (salt).

Equivalent amounts of salt and sodium in differing units (g, mg and mmol)		
Salt (sodium chloride)	Sodium	
grams	mg	mmol
1	400	17.4
5	2000	87
5.75	2300	100
teaspoon	~2300	~100

The table provides approximate equivalent amounts of sodium and salt.
- Approximately 30% of hypertension prevalence can be attributed to high dietary sodium, which could result in hypertension in 400 to 500 million people, worldwide [5–7]. The evolving definition of hypertension includes all people with a usual systolic BP of ≥140 mmHg or diastolic ≥90 mmHg and those at high risk for CVD with a usual systolic BP of ≥130 mmHg [8]. Reductions in dietary sodium can have a larger or smaller impact on hypertension prevalence, depending on the population distribution of sodium intake, BP distribution, extent of decrease in dietary sodium and prevalence of other causes of increased BP [7]. The INTERSALT study and animal studies indicate high dietary sodium consumption may have a substantively larger life course impact on BP than is identified in the currently available relatively short-term sodium reduction trials and suggest that a component of the increased BP may be irreversible [9, 10]. Hence, the adverse effects may be greater than currently predicted and greater emphasis may be needed before permanent harm occurs in younger people.
- The Global Burden of Disease Study estimated that in 2019 there were over 1.8 million deaths, and over 44 million disability-adjusted life years lost (including 40.5 million DALYs from CVD, including stroke), as a result of excess dietary sodium consumption [11].
- In populations that consume less than 1000 mg sodium (2.5 gm salt) per day, hypertension is rare [12–14].
- A substantial proportion of BP-related disease occurs in people who have an average BP below the levels used to identify hypertension [5, 15]. Hence sodium reduction is relevant both for people with hypertension and those with a BP above the optimal level but not yet hypertensive.
- Meta-analyses of randomized controlled trials demonstrate that reducing dietary sodium intake decreases BP in both those with and without hypertension, in children and in adults, and in all ethnic groups [16–20]. The association between BP and dietary sodium intake is approximately linear above 800 mg (2 gm salt) per day.
- Individuals can be more or less prone to the adverse effects of sodium (‘salt sensitivity’) on a genetic, physiological or pathophysiological basis (e.g., primary hyperaldosteronism). There is a steeper sodium BP dose response slope in those who have hypertension, are older, or are of black African ancestry [17].
- A meta-analysis of randomized controlled trials showed a linear decrease in CVD with reductions in sodium between 4100 mg (10.25 gm salt) and 2300 mg (5.75 gm salt) per day [19]. Overall, this evidence has been characterized as moderate rather than strong because of an insufficient number of events. However, the one cohort study that the National Academies of Sciences, Engineering, and Medicine viewed as having low bias, found a linear association between sodium intake and mortality with less mortality at sodium intake below 2300 mg (5.75 gm salt) per day than above 3600 mg (9 gm, salt) per day [19, 21]. A more recent meta-analysis of cohort studies, that classified usual sodium intake with multiple 24 h. urine collections, found a direct linear association between sodium intake (1846 to 5230 mg (4.6 to 13.8 g salt) per day) and cardiovascular events [22]. Each 1000 mg (2.5 gm salt) per day increase in sodium excretion was associated with an 18% increase in cardiovascular events [22].
- Other diseases that have been associated with a high sodium intake include gastric cancer (probable procarcinogen) [23, 24], recurrent calcium-oxalate kidney stones [25], osteoporosis [26], obesity [27, 28], Meniere’s disease [29, 30], headache [31], and renal and cardiac damage [16]. The quality of evidence for many of these disease associations is mixed and they are largely based on observational studies in which it is difficult to confirm causality. A variety of pathophysiologic mechanisms (e.g., increased inflammation and generation of reactive substances [32–35]) support the potential for high sodium intake causing a broad range of disease.

## Scientific reviews of the evidence by governmental and nongovernmental health organizations

- Several independent, comprehensive, and unbiased scientific reviews of the evidence conducted by governmental organizations provide recommendations for reduction in dietary sodium (Table 2) [19, 36–39].

**Table 2:** Selected government / governmental organization / multilateral agency recommendations population recommendations for dietary sodium in adults^a^.

Government / governmental organization / multilateral agency recommendations	Dietary sodium recommendations for adults, mg per day (salt g per day)
World Health Organization [38]	<2000 (<5)
India [131]	2000 (5)
United States and Canada (National Academies of Sciences, Engineering, and Medicine) [19]	<2300 (<5.75) for chronic disease risk reduction; adequate intake 1500 (3.75)
China (Healthy China Action Plan https://www.nhc.gov.cn/guihuaxxs/s3585u/201907/e9275fb95d5b4295be8308415d4cd1b2.shtml, accessed July 23, 2021)	2000 (5)
European Union [37]	~ 2000 (5)
Australia and New Zealand [39]	<2000 (<5); adequate intake 460–920 (1.15–2.3)
Russia [132]	<2400 (<6)
United Kingdom (https://pathways.nice.org.uk/pathways/diet/national-policy-on-diet#content=view-node:nodes-reducing-salt-saturated-and-trans-fats, accessed June 18 2021; https://www.nhs.uk/conditions/vitamins-and-minerals/others/, accessed June 18 2021)	<2400 (<6) with the National Institute for Health and Care Excellence indicating an ultimate goal of 1200 (3)
Brazil [133]	<2000 (<5)
South Africa [134]	<2000 (<5)
Nigeria [135]	<2000 (<5)

^a^Several guidelines recommend lower limits for children based on their lower caloric intake or by providing specific lower targets for age categories of children [136].
- Most non governmental health and scientific organizations provide recommendations to reduce dietary sodium (e.g., International Society of Hypertension [40], Chinese Hypertension League [41], British and Irish Hypertension Society [42], Turkish Hypertension Consensus Report [43], European Societies of Hypertension and of Cardiology [44], American College of Cardiology and American Heart Association [45], Japanese Society of Hypertension [46], Brazilian Hypertension Guideline [47] and a broad rage of Canadian Health and Scientific organizations (https://hypertension.ca/wp-content/uploads/2019/01/Sodium-Fact-Sheet-FINAL-Jan-23-2019.pdf). The American Heart Association and the American College of Cardiology - American Heart Association Hypertension Recommendations advise that adults with hypertension should optimally consume less than 1500 mg sodium (3.75 gm salt) per day but that any reduction is beneficial [45].

## Globally, people consume too much sodium

- The average global intake of sodium in adults is estimated to be about 4000 mg per day (salt 10 g per day), with higher intakes in Asia than other regions [48–50]. However, there is uncertainty regarding the exact levels of population sodium intake in many countries because few representative population studies have been based on 24 h. urine collections, the best way of estimating sodium intake [49, 50].
- Only a small portion of dietary sodium intake results from consumption of unprocessed natural foods: <700 mg per day (salt < 1.75 g per day) in a typical mixed paleolithic non-vegetarian diet and <200 mg per day (salt < 0.5 g per day) in a paleolithic vegetarian diet [51].
- In many high-income countries, most of the sodium consumed (70–80%) results from addition of sodium during food manufacturing and during food preparation in fast-food and sit-down restaurants. In many middle- and low-income countries, excessive sodium intake results from ‘discretionary’ addition of sodium, high-sodium sauces and condiments during home cooking and use of saltshakers at the table. However, globalization of the food industry (nutrition transition) is increasing the exposure of populations in middle- and low-income countries to sodium in processed foods [16] [52–55].
- Table 3 provides standardized nomenclature for describing levels of sodium intake recommended by the World Hypertension League, and partner organizations, based on the level of sodium intake recommended by the World Health Organization [12].

**Table 3:** Standardized nomenclature of levels of sodium intake.

Terminology	Dietary Intake, per day		
	Salt (g)	Sodium (mg)	Sodium (mmol)
Recommended	<5	<2000	<87
High	≥5–10	≥2000–4000	≥87–174
Very high	>10–15	>4000–6000	>176–264
Extremely high	>15	>6000	>264

## Reduction in dietary sodium saves lives, health care resources and costs

- Noncommunicable diseases threaten the global economy and economic development. In response, the World Health Assembly supports nine targets for prevention and control of noncommunicable diseases, including a key recommendation to reduce dietary sodium by 30% by 2025 [56].
- Reducing dietary sodium is one of the most impactful and cost-effective mechanisms to improve population health and is one of the World Health Organization’s ‘best buys’ for prevention of chronic disease [57, 58] (https://resolvetosavelives.org/cardiovascular-health/lives-saved-calculator, accessed July 25, 2021).
- A modest 15% reduction in dietary sodium is estimated to prevent 8.5 million deaths over 10 years in 23 developing countries where 80% of chronic disease deaths in developing nations occur [59]. An analysis published in 2019 showed that a 30% reduction of sodium, could save 40 million lives globally within 25 years [60].
- In low- and -middle income countries, programs to reduce dietary sodium were estimated to provide a return on investment of 13–18:1 [61–63].

## Controversies related to dietary sodium reduction are based largely on low quality research

- There are no definitive randomized controlled trials to define the optimum level of sodium intake to reduce mortality and morbidity, which creates controversy for some [64]. Very large, expensive trials of long duration in different populations would be required, and it is difficult for individuals, even in a clinical trial setting, to maintain a substantially reduced sodium diet in the current high sodium food environments [65]. Nevertheless, there is evidence that sodium reduction prevents CVD events in randomized comparisons of those assigned to a dietary sodium reduction behavioral intervention compared with usual care during long-term follow up (trial and post-trial experience) even though optimal levels of sodium intake remain undefined [19, 21, 66].
- Several prospective cohort studies in high profile journals have identified paradoxical J- and U-shaped relationships between sodium intake and CVD events, leading to a controversial conclusion that dietary sodium intake should only be reduced in adults with a very high daily sodium intake (>5000 mg (12.5 gm salt) per day) [64, 67, 68]. These studies have been criticized as having significant methodological limitations that could alter sodium intake disease associations (e.g., inaccurate measurement of baseline sodium intake, residual confounding, reverse causality, inadequate adjustment of confounding factors, inadequate sample sizes, and follow-up duration) [69]. Many of the controversial studies that have identified a paradoxical relationship between sodium intake and CVD have employed spot (single untimed spontaneously voided) urine samples to estimate usual sodium intake. The Kawasaki, and other formulae, used to estimate 24 h sodium intake based on spot urine measurements have been shown to result in biased estimates of sodium intake compared with estimates based on 24 h. urinary collections [50, 70, 71], to result in a spurious J-shaped association between sodium intake and mortality, and to provide an inaccurate representation of the association between dietary sodium and BP [72–74].
- A 2019 report from the U.S. National Academies of Sciences, Engineering, and Medicine confirmed an Agency for Healthcare Research and Quality report that many of the controversial studies had a high risk of bias and stated “the paradoxical J- and U-shaped relationships of sodium intake and CVD and mortality are likely observed because of methodological limitations of the individual observational studies” [19].
- Similarly, international scientific organizations and scientific reviews concluded that low quality research methods and designs were a source of controversy regarding the benefits of reducing the intake of dietary sodium [42, 75–78].
- A major issue is that estimation of dietary sodium intake is challenging because intake varies substantially from day to day, depending on food choice and portion size, as well as random variation [79, 80]. The best feasible estimate of dietary sodium intake for individuals in clinical research is based on multiple, carefully collected, 24 h urines on nonconsecutive days, but few studies have used this methodology [77, 81]. Instead, most studies use methods that are very inaccurate with both systematic and random error in assessing usual sodium intake [19, 69, 77, 81–84].
- Other studies with controversial findings have used dietary recall or food frequency questionnaire methods for estimation of 24 h dietary sodium intake. These are not recommended for this purpose because they are known to underestimate dietary sodium intake and to be unreliable for assessing an individual’s sodium intake [83, 84].
- One study found that a single 24 h. estimate of usual sodium intake had a spurious J curve association with cardiorenal outcomes that became linear when multiple 24 h urine assessments defined usual sodium intake [85].

## Resources for keeping up to date on the evolving evidence on dietary sodium and how to reduce dietary sodium

- The World Hypertension League, along with other national and international partners and the Journal of Human Hypertension, have developed multiple mechanisms to ensure that the evidence on dietary sodium intake is maintained up to date.
- The ‘science of salt’, a regularly updated critical appraisal of research evidence related to dietary sodium measurement and consumption, clinical consequences, and effectiveness of programs to reduce dietary sodium intake has been published in the Journal of Clinical Hypertension from 2013 until 2020 and more recently in the Journal of Human Hypertension [49, 86–92] (https://www.georgeinstitute.org/projects/science-of-salt-weekly, accessed June 19, 2021).
- Resolve To Save Lives maintains a website that includes best practices in dietary sodium reduction and an updated annotated bibliography which summarizes important evidence on sodium intake, reduction strategies, and measurement (https://resolvetosavelives.org/cardiovascular-health/sodium, and https://linkscommunity.org/toolkit/salt-reduction; accessed July 18, 2021).
- The Nourishing Framework provides regular updates to governmental policies to promote healthier nutrition including reducing dietary sodium (https://www.iccp-portal.org/system/files/resources/PPA_Nourishing_A5%2520leaflet_web%2520FINAL.pdf, accessed June 18, 2021).
- The Centre for Disease Control and Prevention (USA) has a CDC salt bites newsletter that provides updates on sodium reduction research and activities (https://www.cdc.gov/salt/index.htm).
- The WHO Collaborating Centre on Population Salt Reduction at the George Institute for Global Health also regularly reviews national salt reduction activities around the world [93] and features a regular newsletter updating sodium reduction activities and science (https://www.whoccsaltreduction.org/).
- The Centre for Science in the Public Interest hosts a sodium listserv communications group (subscribe by contacting tschwab@cspinet.org).
- World Action on Salt, Sugar and Health (WASSH) provides regular updates on publications and worldwide salt reduction activities (http://www.worldactiononsalt.com/news/salt-in-the-news/2021/).
- The World Health Organization and the Pan American Health Organization provide updated national policy actions on dietary sodium reduction (https://extranet.who.int/nutrition/gina/es/scorecard/sodium and https://www.paho.org/en/noncommunicable-diseases-and-mental-health/noncommunicable-diseases-and-mental-health-data-29, respectively, accessed August 16, 2021).

## Multicomponent comprehensive policies can be effective in reducing dietary sodium intake and have been associated with reductions in BP and CVD [94, 95]

- The World Health Organization technical package for dietary sodium reduction ‘SHAKE’ is based on
  - Surveillance: to measure and monitor the amount of sodium consumed, the main dietary sources of sodium and the amount of sodium in specific foods.
  - Harnessing (through policies that include regulations) the food industry to reduce the amount of sodium added in food processing including the setting of targets and timelines for sodium content of foods [96, 97].
  - Adopting front of package food labels and implementing strategies to reduce misleading marketing of high sodium foods.
  - Knowledge enhancement to empower individuals to eat less sodium.
  - Environmental changes through healthy food procurement policies.

- The World Health Organization has developed global benchmarks for the sodium content of packaged foods (https://www.who.int/publications/i/item/9789240025097, accessed Aug 16, 2021), as has the Food and Drug Administration (FDA United States, https://www.fda.gov/media/98264/download, accessed Oct 19, 2021) and the Pan American Health Organization has updated its regional benchmarks for sodium content of packaged foods [98, 99].
- The Pan American Health Organization [100] and the World Health Organization Regional Office for Europe (https://www.euro.who.int/__data/assets/pdf_file/0006/457611/Accelerating-salt-reduction-in-Europe.pdf), accessed August 2, 2021) also have useful technical resources for reducing dietary sodium [101].
- Resolve to Save Lives has a comprehensive framework for dietary sodium reduction programs that includes resources, implementation tools and examples of successful interventions (https://linkscommunity.org/toolkit/sodium-framework, accessed Nov 25, 2021).
- As of 2020, 96 countries had national strategies to reduce dietary sodium intake [93]. A recent systematic review of sodium reduction found that 4 population-based interventions had reduced average sodium intake levels by 800 or more mg (>2 gm salt)/day (Argentina, China, South Korea, Turkey) and 9 countries had reduced between 400 and 800 mg (1–2 gm salt)/day [93]. Gradual (over a few months) but substantial reductions in sodium of processed foods can be made without altering the perceived taste of food [102].
- Population-based sodium reduction interventions in Japan, Finland and the United Kingdom have also been associated with reduction in BP and CVD [103–107].
- Clinical trials longer than 5 weeks indicate reducing dietary sodium to 2300 mg (5.75 g salt) /day in older adults is feasible and could reduce mortality from stroke by 39% and ischemic heart disease by 30% [108]. A good practical example of the successful implementation of a salt intake reduction program on a national level is Japan, where such an intervention was associated with a dramatic reduction in stroke mortality [107].
- Governments in more countries should take action to develop and implement multi-sectoral national strategies based on the WHO SHAKE technical package to reduce sodium consumption using implementation research methodology [95, 109, 110].
- Broad policies to reduce dietary sodium and consumption of ultra processed foods to improve nutrition (e.g., mandatory sodium targets, front of pack warning labels, marketing restrictions especially to children, healthy public food procurement, and fiscal measures (i.e., taxes)) are believed to be important to reduce population sodium intake [95, 109].
- Industry-based voluntary approaches to reduce the addition of sodium during food processing have a long history of being ineffective unless they are coupled with strong government oversight and close monitoring [111]. Government-led regulated approaches may be more effective [112].
- Public education (particularly through mass media campaigns) and behavior change interventions (e.g., using a COMBI framework) are likely important as part of a broader strategy, especially where discretionary sodium is the major dietary source [113–116]. The use of social marketing strategies and ‘whole of society’ approaches may be beneficial to change social norms and behaviours related to the use of discretionary sodium [113, 117].
- In a recent randomized controlled trial, replacing regular salt with a reduced-sodium salt (where 25% of the sodium was replaced with potassium) in adults with stroke or at high risk for stroke reduced the risk of stroke (14%), cardiovascular events (13%) and premature death (12%) without any evidence of an increased risk of hyperkalemia [118]. Reduced sodium salt can be considered as part of a population sodium reduction strategy and is likely to be most effective in countries where discretionary salt constitutes a significant source of dietary sodium (annotated bibliography https://linkscommunity.org/toolkit/sodium-reduction-an-annotated-bibliography#_Toc14352403, accessed July 25, 2021) [119–124]. Reduced sodium salts and condiments may also help to reduce sodium intake from packaged foods, restaurant foods and discretionary use [125]. Regulatory changes, such as making labeling of potassium additives in processed food products more consumer friendly, may help (e.g., labeling potassium additives as potassium or potassium salt versus potassium chloride).
- Integrating efforts to reduce dietary sodium with those to optimize dietary potassium and iodine through salt fortification are important to enhance health [119, 120, 126, 127].
- Close monitoring of sodium intake, sources of sodium in the diet, sodium levels in foods, as well as knowledge, attitudes and behaviours of the public are essential components of sodium reduction programs [100, 109].

## National hypertension, CVD, nutrition, and health organizations

Hypertension, CVD, nutrition, and health organizations have important roles in research, interpretation of research, education, and advocacy. We call on these organizations to:

- Provide organizational support for this Call to Action by contacting the World Hypertension League at whleague17@gmail.com. An updated list of supporting organizations will be maintained until 2025.
- Promote research, presentations and publications on high quality research related to dietary sodium emphasizing the importance of high-quality research methodology, data that are in the public domain and where interpretation is free of commercial interest.
- Educate members on the health risks of high dietary sodium and how to reduce sodium intake.
- Broadly disseminate relevant information on dietary sodium integrated with other healthy nutrition and physical activity advice to the public and patients.
- Educate policy and decision makers on the health benefits of lowering BP among normotensive and hypertensive people, regardless of age.
- Advocate for policies and regulations that will contribute to population-wide reductions in dietary sodium, possibly in collaboration with other health advocacy groups. The World Health Organization has released a Sodium Country Score Card to track governmental progress to reduce dietary sodium that can be used by health and nutrition organizations and experts in advocacy. (https://extranet.who.int/nutrition/gina/es/scorecard/sodium accessed July 18, 2021).
- Provide opportunities for members to be involved in advocacy. Reach the public and policy makers by promoting and advocating through media releases and social media campaigns on dietary sodium reduction.
- Promote coalition building, increase organizational capacity for advocacy, and develop advocacy tools to promote civil society actions.
- Be cautious about the role of low-quality research, research from domains that are not publicly accessible to be independently validated, and of investigators with commercial conflicts of interest in generating controversy related to dietary sodium reduction.
- Global networks of concerned health care professionals and scientists have formed to help support reductions in dietary sodium. World Action on Salt, Sugar and Health (WASSH) sponsors World Salt Awareness Week annually during the second week of March (www.worldactiononsalt.com/, accessed June 12, 2021). Other organizations with a similar goal include the European Salt Action Network (euro.who.int/en/health-topics/disease-prevention/nutrition/policy/member-states-action-networks/reducing-salt-intake-in-the-population), WHO Collaborating Centre on Population Salt Reduction at the George Institute for Global Health (https://www.georgeinstitute.org/projects/world-health-organization-collaborating-centre-for-population-salt-reduction-who-cc-salt, accessed July 18, 2021), and Action on Salt (http://www.actiononsalt.org.uk/, accessed June 12, 2021).
- Resolve to Save Lives, a global initiative to save 100 million lives in 30 years, has reducing dietary sodium as one of its four pillars [60].

## Public health dietary sodium research priorities

Research is urgently required to accelerate the reduction of dietary sodium in populations. Priorities include research to:

- Better define optimal policies and interventions for reducing dietary sodium in populations including discretionary sodium, sodium from street foods, sodium from packaged foods, and sodium from restaurants. This research is needed in a wide variety of settings and cultures to better understand the obstacles and facilitators to dietary sodium reduction programs.
- Better define optimal interventions for reducing dietary sodium in individuals including discretionary sodium, sodium from street foods, sodium from packaged foods, and sodium from restaurants.
- Accelerate the uptake of best practices in sodium reduction particularly in low- and middle-income countries [128, 129].
- Develop more rapid, feasible and accurate methods to assess individual and population average sodium intake, sources of dietary sodium and levels of sodium in specific foods.
- Better define potential interactions between dietary sodium and potassium in causing disease.
- Implement large scale randomized controlled trials to define optimal levels of sodium and potassium intake in the general population to prevent disease, if feasible designs can be developed.
- Implement large scale randomized controlled trials to assess long term health and common non-CVD diseases reported to be associated with high sodium intake if feasible designs can be developed.
- Define the role of salt (consumed in excess) as a vehicle for providing nutrients that are deficient in the diet (e.g., iodine, fluoride, folate).
- Better identify individuals more or less prone to adverse health consequences from dietary sodium (‘salt sensitivity’).
- Explore the intake of sodium and vulnerability to and complications from COVID -19 infection.
- Uncover the causes and solutions for misinformation on dietary sodium, the role of low-quality research and the role of commercial conflicts of interest in hindering dietary sodium reduction programs.

## World Hypertension League Actions

- The World Hypertension League, Resolve to Save Lives and the International Society of Hypertension have led the development of this fact sheet and call to action targeted at hypertension, cardiovascular, nutrition and health experts and scientists and their organizations to support achievement of the WHO recommended sodium intake levels.
- The World Hypertension League has developed the Graham MacGregor Award and Excellence Awards to recognize organizations and individuals who have contributed to efforts to reduce dietary sodium at the population level. (http://www.whleague.org/index.php/news-awards-recognition, accessed June 1, 2021).
- Assisting the global and national efforts to reduce dietary sodium is a top priority of the World Hypertension League.
